# Correlation of Subclinical Hypothyroidism With Polycystic Ovary Syndrome (PCOS)

**DOI:** 10.7759/cureus.8142

**Published:** 2020-05-15

**Authors:** Murk Fatima, Sofia Amjad, Habiba Sharaf Ali, Tousif Ahmed, Shabnam Khan, Marvi Raza, Mahreen Inam

**Affiliations:** 1 Physiology, Ziauddin University, Karachi, PAK; 2 Obstetrics and Gynaecology, Ziauddin University, Karachi, PAK; 3 Physiology, Ziauddin Hospital, Karachi, PAK; 4 Anatomy, Ziuaddin University, Karachi, PAK; 5 Pathology, Ziauddin University, Karachi, PAK

**Keywords:** polycystic ovary syndrome, subclinical hypothyroidism, insulin resistance

## Abstract

Aim

The correlation of subclinical hypothyroidism (SCH) and polycystic ovary syndrome (PCOS) is a still insufficiently explored entity. The aim of this study was to determine the correlation between SCH and PCOS along with the impact of SCH on metabolic and hormonal parameters in women with PCOS.

Methodology

This cross-sectional study was conducted at the Gynecology Outpatient Department of Ziauddin Hospital Kemari, Karachi, Pakistan, from June 2019 to December 2019. A total of 90 diagnosed cases of PCOS were enrolled in the study. A non-probability consecutive sampling technique was used. After taking informed consent, participants were evaluated through clinical interviews, a questionnaire, and anthropometric measurements. The participants underwent the following assessments, i.e., transabdominal ultrasonography, hormonal profile (free testosterone, follicle-stimulating hormone, luteinizing hormone), and fasting blood sugar. Participants were divided into two groups based on thyroid-stimulating hormone (TSH) into the euthyroid group and subclinical hypothyroid (SCH) group. The Mann-Whitney test was used for comparing the two groups.

Results

Our results showed a significant difference in weight, body mass index (BMI), insulin, homeostatic model assessment of insulin resistance (HOMA-IR), and TSH were found in the SCH group as compared to the euthyroid group. A significant correlation of TSH with waist-hip ratio (WHR), weight, body mass index (BMI), insulin, and the homeostatic model assessment of insulin resistance (HOMA-IR) in PCOS patients.

Conclusion

This study showed a significant correlation of subclinical hypothyroidism with polycystic ovary syndrome. We found subclinical hypothyroidism may aggravate the insulin resistance; therefore, PCOS patients must be screened with a thyroid profile.

## Introduction

Polycystic ovary syndrome (PCOS) is an endocrine disease of females that commonly presents during reproductive life [1]. The pathophysiology of this disease mainly includes chronic anovulation, hyperandrogenemia, and insulin resistance (IR) which may present clinically as abnormal uterine bleeding, hirsutism, infertility, etc. This syndrome is diagnosed by the presence of two of the following three criteria: oligo- or anovulation, clinical and/or biochemical hyperandrogenism, and a finding of polycystic ovaries by ultrasound with the exclusion of other causes [2].

Disorders of the thyroid gland are frequently encountered in patients with PCOS among which subclinical hypothyroidism (SCH) is present in 5% - 10% of PCOS patients [3]. SCH represents a milder form of hypothyroidism that does not present with overt clinical symptoms but may contribute to subfertility and adverse pregnancy outcomes. It is characterized biochemically by an elevated thyroid-stimulating hormone (TSH) level and normal free thyroxine (FT4). Although most endocrinological references use a cutoff of TSH > 4.5 mIU/L to diagnose SCH [4], some studies have reported adverse pregnancy outcomes with TSH levels > 2.5 mIU/L [5]. Hence, the latter is considered a more appropriate cutoff level for patients with infertility or anticipating pregnancy.

Thyroid hormones may also act as insulin agonists in muscle and as antagonists in the liver so a deficiency of thyroid hormones may lead to a decrease in glucose production and utilization. Thus, some authors have considered IR, which has been considered to be the principal factor in the pathogenesis of PCOS, as a consequence of hypothyroidism [6]. It may also increase the conversion of androstenedione to testosterone, affect the gonadal function, and lead to anovulatory cycles [7]. On the other hand, thyroid hormone replacement therapy in PCOS women also leads to a reduction in serum androgen levels, as well as an improvement in the appearance of the polycystic ovaries. Based on the association of thyroid function, obesity, IR, and reproduction, it is conceivable that SCH may influence the presence of PCOS and its related features. However, due to little information on whether SCH aggravates the severity of metabolic and hormonal alterations of women that suffer from PCOS, this study was conducted to evaluate the impact of SCH on PCOS patients. The objective of this study was to evaluate the clinical, hormonal, and metabolic variables of PCOS patients and its association with concurrent SCH.

## Materials and methods

This cross-sectional study was approved by the research ethics board of Ziauddin University (ref. #0910319MFPHY).

A total of 90 women who were diagnosed with PCOS, according to the Rotterdam European Society for Human Reproduction and Embryology/American Society for Reproductive Medicine (ESHRE/ASRM) Consensus Criteria 2003, were included in the study at the Gynecological Outpatient Department (OPD) at Ziauddin Hospital, Kemari, Karachi, Pakistan, from June 2019 to December 2019. It was carried out in women between 16 to 35 years [8].

After taking informed consent all participants were interviewed on an individual basis about the pattern of the menstrual cycle, infertility, weight problem, past diagnosis or treatment of PCOS, or any other illnesses and hirsutism. Furthermore, no participant received any form of thyroid treatment within six months of the start of the study. Weight and height were measured by calibrated instruments, and ultrasonographic findings of participants were recorded. TSH was determined using a thyroid-stimulating hormone Enzyme-linked Immunosorbent Assay (ELISA) Kit (MyBioSource, San Diego, CA, USA) with a reference range from 0.05 mIU/L - 30 mIU/L, and analytical sensitivity of 0.023 mIU/L. The procedure of the manufacturer's protocol of the kit was followed. Serum samples of patients were added to the appropriate microtiter plate wells pre-coated with a biotin-conjugated antibody specific to thyroid-stimulating hormone (TSH). Next, avidin conjugated to horseradish peroxidase (HRP) was added to each microplate well and incubated. After tetramethylbenzidine (TMB) substrate solution was added, only those wells that contained thyroid-stimulating hormone (TSH), biotin-conjugated antibody, and enzyme-conjugated avidin exhibited a change in color. The enzyme-substrate reaction was terminated by the addition of sulphuric acid solution and the color change was measured spectrophotometrically at a wavelength of 450 nm ± 10 nm.

Patients were divided into two groups: the SCH group with a TSH > 2.5 mIU/L and the euthyroid group with a TSH < 2.5 mIU/L [9]. We compared both groups based on mean age, body mass index (BMI), fasting plasma glucose (FPG), and fasting insulin. Fasting serum insulin was estimated by the Siemens IMMULITE® 1000 chemiluminescence immunometric assay (Siemens Medical Solutions USA, Inc., Malvern, PA, USA). This assay utilized one anti-insulin antibody for solid phase (microtiter wells) immobilization and another anti-insulin antibody in the antibody-enzyme (horseradish peroxidase) conjugate solution. The standards and test specimen (serum) were added into insulin antibody-coated microtiter wells. Then, the anti-insulin antibody labeled with horseradish peroxidase (conjugate) was added. After a one-hour incubation at room temperature, the wells were washed with water to remove unbound labeled antibodies. A solution of the chemiluminescent substrate was then added and relative light units (RLU) were read in a luminometer. The intensity of the emitting light was proportional to the amount of enzyme present and was directly related to the amount of insulin in the sample. According to Melmed et al., a fasting insulin level < 25 mIU/L (< 174 pmol/L) was considered normal [10]. Insulin resistance was evaluated according to the homeostasis model assessment (HOMA) using the following formula: HOMA = (fasting insulin mIU/mL * fasting glucose mM)/22.5. Insulin resistance was diagnosed when HOMA was > 2.5 [11].

Statistical analysis

The statistical analysis was performed with the Statistical Package for Social Sciences (SPSS), version 20 (IBM SPSS Statistics, Armonk, NY). For categorical variables, frequencies and percentages were calculated. For numeric variables, the mean, standard deviation (SD), and the median were calculated. The Mann-Whitney test applied for comparing two groups. For finding a correlation, Spearman's correlation was applied.

## Results

A total of 90 women diagnosed with PCOS were recruited for the study. TSH was normal in 59 (65.6%) women, whereas 31 (34.4%) women had SCH. Among the SCH group (n = 31), 19 women (61.3%) were obese and 12 (38.7%) were overweight, according to BMI measurements. Twenty-four women (77.4%) had normal serum insulin levels and seven (22.6%) had raised levels, while a total of 28 (91.3%) women in the SCH group had insulin resistance.

While comparing two groups, we found a significant difference in mean of weight (p = .000), mean BMI (p = .000), mean insulin (p = .002), mean HOMA-IR (p = .000), and mean TSH (p = .000) were found as compared with the euthyroid group, as shown in Table 1. However, no significant difference was found for age, WHR, FBS, hormonal profile (luteinizing hormone (LH), FSH, and testosterone) between the groups.

**Table 1:** Differences in Demographic Characteristics and Laboratory Values Between Women With and Without Subclinical Hypothyroidism in Polycystic Ovary Syndrome *≤ 0.05 p-value. **≤ 0.001 p-value BMI: body mass index; BP-DYS: blood pressure - systolic; BP-SYS: blood pressure - systolic; FBS: fasting blood sugar; FSH: follicle-stimulating hormone; HOMA-IR: homeostatic model assessment of insulin resistance; LH: luteinizing hormone; TSH: thyroid-stimulating hormone; WHR: waist-hip ratio

	Subclinical Hypothyroid (n=31)	Euthyroid (N=59)	P-value
Age	24.6 ± 4.67	23.4 ± 4.54	.228
Weight	82.9 ± 0.81	69.8 ± 9.83	< 0.001 **
Height	5.3 ± .26	5.4 ± .292	< 0.005*
BP.SYS	113.0 ± 6.94	114.3 ± 8.17	.726
BP.DYS	80.6 ± 7.04	79.0 ± 5.125	.05
BMI	32.5 ± 3.75	25.7 ± 3.27	< 0.001**
WHR	5.3 ± 19.	12.4 ± 30.52	< 0.001**
FBS	96.8 ± 12.27	94.7 ± 8.41	.145
Serum insulin	21.0 ± 5.79	14.4 ± 6.87	< 0.001**
HOMA.IR	4.9 ± 1.50	3.2 ± 1.69	< 0.001**
Testosterone	87.2 ± 33.11	84.9 ± 36.57	.875
LH	11.6 ± 5.37	12.3 ± 4.21	.494
FSH	7.6 ± 3.16	6.8 ± 2.85	.252
TSH	3.9 ± 1.09	1.8 ± .38	< 0.001**

While finding correlation of TSH with clinical, metabolic and hormonal parameters, our result showed significant correlation of WHR (p-value = 0.035), weight (p-value < 0.001), BMI (p-value < 0.001), insulin (p-value < 0.001), and HOMA-IR (p-value < 0.001), while there was no correlation of fasting blood glucose level and hormonal levels (LH, FSH, and testosterone) with TSH (Table 2).

**Table 2:** Correlation of Subclinical Hypothyroidism With Clinical, Metabolic, and Hormonal Parameters r_s_: Spearman's rank correlation coefficient *≤ 0.05 p-value **≤ 0.001 p-value BMI: body mass index; FBS: fasting blood sugar; FSH: follicle-stimulating hormone; HOMA-IR: homeostatic model assessment of insulin resistance; LH: luteinizing hormone; TSH: thyroid-stimulating hormone; WHR: waist-hip ratio

CORRELATION		
Clinical, metabolic, and hormonal parameters		TSH
WHR	r_s_	. 223
	P	.035**
Weight	r_s_	.483
	P	< 0.001**
BMI	r_s_	.665
	P	< 0.001 **
LH	r_s_	-.147
	p	.166
FSH	r_s_	.137
	P	.198
Testosterone	r_s_	-.004
	P	.971
Insulin	r_s_	.452
	P	< 0.001 **
HOMA-IR	r_s_	.440
	P	< 0.001 **
FBS	r_s_	.136
	P	.201

## Discussion

PCOS and thyroid pathology, the two endocrine abnormalities, have been connected to each other for quite a long while. In our study, the prevalence of SCH among women with PCOS was considerably higher as compared to previous studies [3, 9, 12]. This might be due to the increased rate of obesity in Pakistani women [13]. Another reason may be due to using a TSH cutoff value > 2.5 mIU/L for SCH. This cutoff value was chosen based on the American Thyroid Association (ATA) consensus guideline (Recommendation 20) that recommends treatment of SCH due to higher reported clinical pregnancy rates and lower miscarriage rates after assisted reproductive treatment (ART) when TSH levels are maintained < 2.5 mIU/L [14].

There was a significant correlation between TSH with weight, BMI, insulin, and HOMA-IR. Duntas et al. mentioned in his research that leptin, an adipocyte hormone, was a major factor linking obesity and thyroid autoimmunity [15]. When TSH binds to the receptor in adipocytes, it stimulates interleukin-6 release from adipocytes and then mediates proliferation, differentiation, and leptin secretion of preadipocytes and adipocytes [16]. The growing evidence also suggests an association of altered thyroid function and obesity causes a lasting state of low-grade inflammation, while Nayak et al. found no association between obesity and subclinical hypothyroidism among PCOS patients [17-18].

**Figure 1:**
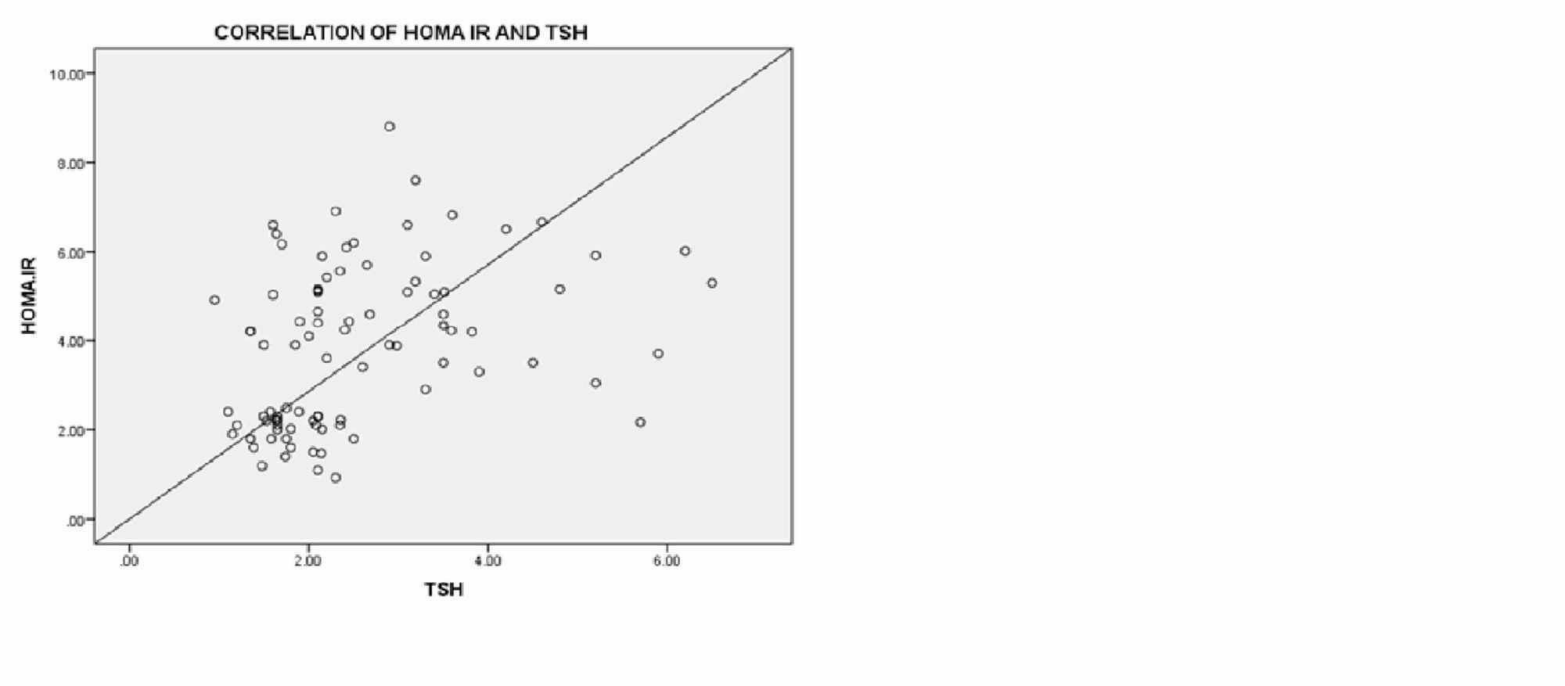
Correlation of HOMA-IR with TSH HOMA-IR: homeostatic model assessment of insulin resistance; TSH: thyroid-stimulating hormone

Our data show a positive correlation of TSH and fasting insulin and HOMA IR as also seen in other studies [19]. Bediway et al. also mentioned TSH had a positive association with HOMA-IR [9]. Figure 1 shows an increase in TSH causing increased insulin resistance.

Increased TSH levels may cause altered adipocyte physiology which causes the decreased release of insulin-sensitizing adipokines, such as adiponectin, that may lead to insulin resistance and associated comorbidities, such as the future development of type II diabetes mellitus (DM) [20].

Some studies did not find an association of TSH with HOMA-IR [12, 21]. We found no correlation of TSH with FBS as in contrast to the Bedaiwy et al. study which showed an association between TSH and FBS [9]. De Medeiros et al. showed normal FBS levels in the SCH group [22].

Our results showed there was a significant difference between euthyroid and SCH in weight, BMI, HOMA-IR, and insulin as in some studies [9, 19, 23]. Trakakis et al. found patients with SCH and PCOS did not differ from PCOS patients with normal thyroid function in terms of BMI, waist/hip circumference, and HOMA-IR [3]. The study by Benetti-Pinto et al. also supported these findings [24]. Enzevaei et al. reported findings contradictory to ours, stating that SCH in PCOS did not have a significant impact on IR [12].

Regarding hormones, no significant difference was found in our study between the levels of LH, FSH, and testosterone in the two groups. Along with it, there was no correlation found between TSH and LH, FSH, and testosterone. Enzaevi et al. reported free testosterone was significantly different in the SCH and euthyroid PCOS groups [12]. Zhang et al. also reported high levels of TSH associated with free testosterone [7].

## Conclusions

PCOS is a multifactorial disorder. SCH has been reported to be more prevalent in PCOS. This study concluded SCH significantly correlated with PCOS patients. A significant effect of SCH on clinical, metabolic, and hormonal characteristics of PCOS patients was seen in our study. This may be due to further aggravation of insulin resistance in PCOS by subclinical hypothyroidism. Hence, screening for a thyroid problem in PCOS patients is recommended, even in the absence of symptoms related to thyroid dysfunction, which may benefit the patient clinically. The limitation of our study that it was a single-centered study so future studies should be carried on large numbers of patients with and without PCOS.
